# Supplementary material for: Asexual reproduction reduces transposable element load in experimental yeast populations
Source: eLife. 2019 Sep 5;8:e48548. doi: 10.7554/eLife.48548 (PMC6783261; doi:10.7554/eLife.48548)
Supplement: Supplementary file 1. [file elife-48548-supp1.docx]

**Element Internal LTR combined boundaries range**
TY1/Copia 5249 338 5925 5425-6425
TY2/Copia 5295 332 5958 5458-6458
TY3/Gypsy 4671 340 5351 4851-5851
TY3_1p/Gypsy 4675 365 5405 4905-5905
TY4/Copia 5484 371 6226 5726-6726
TY5/Copia 4874 251 5376 4876-5876

**Supplementary file 1.** *S. cerevisiae* TY elements and the sizes (in bp) of internal regions and LTRs and the size boundaries used for filtering.
