# Supplementary material for: Asexual reproduction reduces transposable element load in experimental yeast populations
Source: eLife. 2019 Sep 5;8:e48548. doi: 10.7554/eLife.48548 (PMC6783261; doi:10.7554/eLife.48548)
Supplement: Supplementary file 2. — (A) Explored parameter space of the simulations as pertinent for yeast (empirically determined values in bold). Selection_a and selection_b are selection coefficients for linear fitness effects and epistasis, respectively. Lost_TEs refers to the total number of TE lost after 1000 generations (averaged over ten replicates). (B) Explored parameter space for simulations including a modifier allele. Highlighted is the simulation closest to empirical observations. Init_f is the frequency of the modifier at the start of the simulations. Selection_a and selection_b are selection coefficients for linear fitness effects and epistasis, respectively. Lost_TEs refers to the total number of TE lost after 1000 generations (averaged over ten replicates). The bold lines refer to parameter combinations that generate results close to the observed empirical values. [file elife-48548-supp2.docx]

| transposition_rate | exision_rate | selection_a | selection_b | sex_lost_TEs | asex_lost_TEs |
| --- | --- | --- | --- | --- | --- |
| 1.00E-06 | 5.00E-07 | 1.00E-04 | 0 | 6.5 | 2 |
| 1.00E-05 | 5.00E-07 | 1.00E-04 | 0 | 6.7 | 1.5 |
| 1.00E-04 | 5.00E-07 | 1.00E-04 | 0 | 3.5 | -0.8 |
| 1.00E-06 | 1.00E-06 | 1.00E-04 | 0 | 8.1 | 2.2 |
| 1.00E-05 | 1.00E-06 | 1.00E-04 | 0 | 7.8 | 2.3 |
| 1.00E-04 | 1.00E-06 | 1.00E-04 | 0 | 5.7 | -0.1 |
| 1.00E-06 | 5.00E-05 | 1.00E-04 | 0 | 28.6 | 17 |
| 1.00E-05 | 5.00E-05 | 1.00E-04 | 0 | 21.9 | 13.1 |
| 1.00E-04 | 5.00E-05 | 1.00E-04 | 0 | 19.4 | 11 |
| 1.00E-06 | 5.00E-07 | 5.10E-04 | 0 | 6.7 | 1.9 |
| 1.00E-05 | 5.00E-07 | 5.10E-04 | 0 | 6.4 | 1.6 |
| 1.00E-04 | 5.00E-07 | 5.10E-04 | 0 | 4.3 | -0.2 |
| 1.00E-06 | 1.00E-06 | 5.10E-04 | 0 | 9.1 | 2.5 |
| 1.00E-05 | 1.00E-06 | 5.10E-04 | 0 | 8.2 | 2.4 |
| 1.00E-04 | 1.00E-06 | 5.10E-04 | 0 | 6.3 | 0.5 |
| 1.00E-06 | 5.00E-05 | 5.10E-04 | 0 | 22.7 | 14 |
| 1.00E-05 | 5.00E-05 | 5.10E-04 | 0 | 22.6 | 13.6 |
| 1.00E-04 | 5.00E-05 | 5.10E-04 | 0 | 20.6 | 11.3 |
| 1.00E-06 | 5.00E-07 | 1.00E-04 | 0.00039 | 6.5 | 2 |
| 1.00E-05 | 5.00E-07 | 1.00E-04 | 0.00039 | 6.7 | 1.5 |
| 1.00E-04 | 5.00E-07 | 1.00E-04 | 0.00039 | 3.5 | -0.8 |
| 1.00E-06 | 1.00E-06 | 1.00E-04 | 0.00039 | 8.1 | 2.2 |
| 1.00E-05 | 1.00E-06 | 1.00E-04 | 0.00039 | 7.8 | 2.3 |
| 1.00E-04 | 1.00E-06 | 1.00E-04 | 0.00039 | 5.7 | -0.1 |
| 1.00E-06 | 5.00E-05 | 1.00E-04 | 0.00039 | 28.6 | 17 |
| 1.00E-05 | 5.00E-05 | 1.00E-04 | 0.00039 | 21.9 | 13.1 |
| 1.00E-04 | 5.00E-05 | 1.00E-04 | 0.00039 | 19.4 | 11 |
| 1.00E-06 | 5.00E-07 | 5.10E-04 | 0.00039 | 6.7 | 1.9 |
| 1.00E-05 | 5.00E-07 | 5.10E-04 | 0.00039 | 6.4 | 1.6 |
| 1.00E-04 | 5.00E-07 | 5.10E-04 | 0.00039 | 4.3 | -0.2 |
| **1.00E-06** | **1.00E-06** | **5.10E-04** | **0.00039** | **9.1** | **2.5** |
| 1.00E-05 | 1.00E-06 | 5.10E-04 | 0.00039 | 8.2 | 2.4 |
| 1.00E-04 | 1.00E-06 | 5.10E-04 | 0.00039 | 6.3 | 0.5 |
| 1.00E-06 | 5.00E-05 | 5.10E-04 | 0.00039 | 22.7 | 14 |
| 1.00E-05 | 5.00E-05 | 5.10E-04 | 0.00039 | 22.6 | 13.6 |
| 1.00E-04 | 5.00E-05 | 5.10E-04 | 0.00039 | 20.6 | 11.3 |

**Supplementary file 2A.** Explored parameter space of the simulations as pertinent for yeast (empirically determined values in bold). Selection_a and selection_b are selection coefficients for linear fitness effects and epistasis, respectively. Lost_TEs refers to the total number of TE lost after 1000 generations (averaged over ten replicates).

| init_f | selection_a | selection_b | sex_lost_TEs | asex_lost_TEs |
| --- | --- | --- | --- | --- |
| 0.01 | 2.00E-04 | 0 | 0.6 | 1.2 |
| 0.01 | 3.00E-04 | 0 | 0.7 | 2.7 |
| 0.01 | 4.00E-04 | 0 | 0.6 | 5.3 |
| **0.01** | **0.000425** | **0** | **0.9** | **6.2** |
| **0.01** | **0.00045** | **0** | **0.7** | **6.6** |
| **0.01** | **0.000475** | **0** | **0.8** | **10.3** |
| **0.01** | **5.00E-04** | **0** | **1** | **9.9** |
| 0.01 | 5.00E-04 | 1.00E-06 | 0.3 | 0.3 |
| 0.01 | 1.00E-03 | 1.00E-06 | 0.6 | 0.9 |
| 0.01 | 2.00E-03 | 1.00E-06 | 1.3 | 5.1 |
| 0.01 | 2.00E-04 | 1.00E-05 | 1.3 | 15.6 |
| 0.01 | 3.00E-04 | 1.00E-05 | 1.2 | 17 |
| 0.01 | 4.00E-04 | 1.00E-05 | 2.2 | 20.1 |
| 0.01 | 5.00E-04 | 1.00E-05 | 2.2 | 22.7 |
| 0.1 | 2.00E-04 | 0.00E+00 | 3.4 | 7.6 |
| 0.1 | 3.00E-04 | 0.00E+00 | 4 | 12.6 |
| 0.1 | 4.00E-04 | 0.00E+00 | 4.9 | 16.9 |
| 0.1 | 5.00E-04 | 0 | 6.4 | 20 |
| 0.1 | 2.00E-04 | 1.00E-05 | 7.6 | 22.4 |
| 0.1 | 3.00E-04 | 1.00E-05 | 8.8 | 23.5 |
| 0.1 | 4.00E-04 | 1.00E-05 | 10.6 | 24.9 |
| 0.1 | 5.00E-04 | 1.00E-05 | 11.9 | 26.1 |

**Supplementary file 2B.** Explored parameter space for simulations including a modifier allele. Highlighted is the simulation closest to empirical observations. Init_f is the frequency of the modifier at the start of the simulations. Selection_a and selection_b are selection coefficients for linear fitness effects and epistasis, respectively. Lost_TEs refers to the total number of TE lost after 1000 generations (averaged over ten replicates). The bold lines refer to parameter combinations that generate results close to the observed empirical values.
